# Qualitative Study of an mHealth Mindfulness intervention with Caregivers of Older Adults with MCI or Dementia

**DOI:** 10.1093/geroni/igab046.2969

**Published:** 2021-12-17

**Authors:** Danielle Llaneza, XinQi Dong, Paul Duberstein, Elissa Kozlov

**Affiliations:** 1 Rutgers University, New Brunswick, New Jersey, United States; 2 Rutgers University, Rutgers Institute for Health, New Jersey, United States; 3 Rutgers University School of Public Health, Piscataway, New Jersey, United States; 4 Rutgers University, Rutgers University, New Jersey, United States

## Abstract

Caregivers of patients with dementia experience high levels of emotional distress. mHealth interventions have the potential to feasibly address some needs of caregivers and reduce stress. This qualitative research study of (n = 15) caregivers of patients with dementia explored caregivers’ experience using a mindfulness meditation mobile application. The qualitative interviews were guided and structured to allow participants to share their perceived benefits, drawbacks, likes, and dislikes of using mHealth strategies to manage stress and anxiety. We asked about the caregivers' experience with mindfulness before the study, use of the app, their positive/helpful and negative/unhelpful app experiences, the perceived value of the app, and potential enhancements of the app. Caregivers reported that the app was easy to use, engaging and that there were many perceived benefits. They also noted multiple barriers to using the app including time constraints and implementation of mindfulness techniques outside of direct app use. Most of the caregivers recommended using the app to increase knowledge of mindfulness and to reduce stress. Our findings support the growing body of literature on the practical use of mHealth strategies for caregivers. Future work should address the perceived barriers caregivers encounter when using mHealth strategies.

